# Exercise-induced β2-adrenergic Receptor Activation Enhances the Antileukemic Activity of Expanded γδ T-Cells via DNAM-1 Upregulation and PVR/Nectin-2 Recognition

**DOI:** 10.1158/2767-9764.CRC-23-0570

**Published:** 2024-05-13

**Authors:** Forrest L. Baker, Kyle A. Smith, Preetesh L. Mylabathula, Tiffany M. Zúñiga, Douglass M. Diak, Helena Batatinha, Grace M. Niemiro, Michael D. Seckeler, Charles R. Pedlar, Daniel P. O'Connor, Jamie Colombo, Emmanuel Katsanis, Richard J. Simpson

**Affiliations:** 1 School of Nutritional Sciences and Wellness, University of Arizona, Tucson, Arizona.; 2 Department of Pediatrics, University of Arizona, Tucson, Arizona.; 3 The University of Arizona Cancer Center, Tucson, Arizona.; 4 Department of Pediatrics (Cardiology), University of Arizona, Tucson, Arizona.; 5 Faculty of Sport, Health and Applied Performance Science, St. Mary's University, London, United Kingdom.; 6 Department of Health and Human Performance, University of Houston, Houston, Texas.; 7 Department of Immunobiology, University of Arizona, Tucson, Arizona.; 8 Department of Medicine, University of Arizona, Tucson, Arizona.; 9 Department of Pathology, University of Arizona, Tucson, Arizona.

## Abstract

Exercise mobilizes cytotoxic lymphocytes to blood which may allow superior cell products to be harvested and manufactured for cancer therapy. Gamma-Delta (γδ) T-cells have shown promise for treating solid tumors, but there is a need to increase their potency against hematologic malignancies. Here, we show that human γδ T-cells mobilized to blood in response to just 20 minutes of graded exercise have surface phenotypes and transcriptomic profiles associated with cytotoxicity, adhesion, migration, and cytokine signaling. Following 14 days *ex vivo* expansion with zoledronic acid and IL2, exercise mobilized γδ T-cells had surface phenotypes and transcriptomic profiles associated with enhanced effector functions and demonstrated superior cytotoxic activity against multiple hematologic tumors *in vitro* and *in vivo* in leukemia-bearing xenogeneic mice. Infusing humans with the β1+β2-agonist isoproterenol and administering β1 or β1+β2 antagonists prior to exercise revealed these effects to be β2-adrenergic receptor (AR) dependent. Antibody blocking of DNAM-1 on expanded γδ T-cells, as well as the DNAM-1 ligands PVR and Nectin-2 on leukemic targets, abolished the enhanced antileukemic effects of exercise. These findings provide a mechanistic link between exercise, β2-AR activation, and the manufacture of superior γδ T-cell products for adoptive cell therapy against hematologic malignancies.

**Significance:**

Exercise mobilizes effector γδ T-cells to blood via β2-adrenergic signaling which allows for generation of a potent expanded γδ T-cell product that is highly cytotoxic against hematologic malignancies.

## Introduction

Physical activity and structured exercise are known to reduce cancer risk and progression (1, 2). This is linked to shifts in hormone levels, reduced adiposity, lowered systemic inflammation, and improvements in immune function (3–5). Every bout of moderate to vigorous intensity exercise dramatically increases circulating catecholamine levels, which drive the mobilization of highly cytotoxic lymphocytes to the blood compartment and coupled with the release of myokines facilitates their ability to infiltrate tumors (6). Considering the significant role that exercise-mobilized lymphocytes can play in strengthening immune surveillance and restraining tumor development, we have sought to investigate whether they may be more suitable candidates for adoptive cell therapies (7).

Among the various subsets of effector lymphocytes that exhibit high responsiveness to exercise, γδ T-cells appear to be a promising option for the treatment of both solid and hematologic malignancies (8–12). γδ T-cells, which represent about 5% of circulating lymphocytes, recognize a broad range of tumors in a non–MHC-restricted manner via their γδ-TCR or natural killer (NK)-cell-like activating receptors such as natural killer group 2D (NKG2D) DNAX-activating molecule (DNAM-1), and TNF-related apoptosis-inducing ligand (TRAIL; ref. 13). They are also capable of direct anti-tumor immunity through the release of cytotoxic granules, or indirectly through the production of effector cytokines such as IFNγ and TNFα. A subset of γδ T-cells, Vγ9Vδ2 T-cells, can be stimulated and expanded with aminobisphosphonates, such as Zoledronate (ZOL), and the *ex vivo* expansion of these cells with ZOL+IL2 has been shown to induce cytotoxic effects against many hematologic malignancies and solid tumors in both preclinical and clinical trials (14–18). There is, however, a critical need to increase the potency of expanded γδ T-cells, particularly against hematologic malignancies. For instance, the adoptive transfer of *ex vivo* expanded γδ T-cell has been shown to improve disease progression but failed to improve overall survival (OS) in multiple cancers (19–25). The results were similar when ZOL was used to promote the *in vivo* expansion of γδ T-cells, with little to no effect in most patients (26–32). Critically, approximately 58% of patients receiving *in vivo* ZOL failed to reach γδ T-cell numbers associated with increased OS following allogeneic hematopoietic cell transplantation. Moreover, *ex vivo* expansion has sometimes been unsuccessful due to low numbers of γδ T-cells in circulation (33, 34). We have shown that a single bout of exercise in humans mobilizes γδ T-cells to the peripheral blood and augments their *ex vivo* expansion and anti-tumor activity against a range of hematologic tumors *in vitro* (10). We also found an upregulation of key activating receptors on γδ T-cells expanded after exercise, and antibody blocking of NKG2D abrogated the augmented cytotoxic effects of exercise against a multiple myeloma (U266) but not a chronic myeloid leukemia (K562) cell line (10).

In the current study, we built on these findings by showing that γδ T-cells mobilized with exercise have transcriptomic profiles associated with cytotoxicity, adhesion, migration, and cytokine signaling, and that their increased cytotoxicity against K562 leukemic cells is dependent on an upregulation of DNAM-1 at the cell surface, allowing them to kill K562 cells via poliovirus receptor (PVR) and Nectin-2. The increased anti-leukemic effects of exercise were replicated when γδ T-cells were collected from the blood of humans during isoproterenol [ISO; a synthetic β1+β2-AR (adrenergic receptor) agonist] infusion. In addition, the mobilization and enhanced expansion of γδ T-cells were abolished after administering a β1+β2-AR but not a β1-AR antagonist prior to exercise. These findings provide a mechanistic link between exercise, β2-AR activation, and the manufacture of enhanced γδ T-cell products for adoptive cell therapy against hematologic malignancies.

## Materials and Methods

### Participants

We recruited 26 (9 female) healthy, physically active adults to participate in this study. 10 participants (height: 174 ± 10.1 cm, body mass: 69.2 ± 9.1 kg; age: 29.6 ± 6.5 years, female: *n* = 3) completed the exercise only trials, 9 participants (height: 175.7 ± 7.9 cm, body mass: 75 ± 6.9 kg; age: 28.3 ± 5.4 years, female: *n* = 3) completed the randomized placebo-controlled cross-over trial involving exercise and beta blockers, and 7 participants (height: 173 ± 11.5 cm, body mass: 68.2 ± 11.9 kg; age: 26.8 ± 5.4 years, female: *n* = 2) completed the ISO infusion experiments. Participants were excluded if they were current or recent (within 6 months) users of tobacco products, physically inactive [physical activity readiness (PAR) score <4], obese [body mass index ≥30 kg/m^2^ or waist girth >102 cm (men) and 88 cm (women)], pregnant, or had any illness, disease, or disorder that could affect the immune system. Enrolled participants were not taking medications (except for oral contraceptives for some female participants) and were classified as “low risk” for graded exercise testing in accordance with ACSM/American Heart Association criteria. Participants were required to abstain from alcohol, caffeine, and physical activity 24 hours prior to trials, as well as eliminate vitamin/mineral supplementation and medications that could modulate the immune system at least 4 weeks prior to involvement in the study. Adherence to the pretest guidelines were confirmed verbally with the participants prior to their arrival at the laboratory.

### Experimental Design

All laboratory visits occurred between 7:00 am and 10:00 am and, when multiple visits were required, these were separated by 1–3 weeks. Participants were required to fast overnight (8–12 hours), abstain from alcohol, and refrain from vigorous activities for 24 hours prior to each visit. The study was conducted in accordance with the Declaration of Helsinki and approval from the Human Subjects Protection Program at the University of Arizona (Tucson, AZ; #1801161041 and #1711017841), with written consent from all participants.

#### Standard Exercise Protocol

Participants completed two exercise trials performed at least a week apart, the first being a maximal exercise test to determine 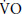
_2max_ (44.9 ± 8.7 mL/kg/minute), and the second being a steady-state or graded bout of exercise up to intensities equivalent to 80% 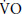
_2max_ on an indoor cycling ergometer, as described previously (10, 35). Heart rate, electrocardiogram (ECG) activity, and respiratory gas exchange were recoded continuously throughout the test (Cosmed CPET). Prior to exercise (REST) and during the last 3–5 minutes of the exercise bout (EX), blood samples were collected from an indwelling catheter into vacuum-sealed tubes containing EDTA or citric acid, sodium citrate, and dextrose (ACD; Becton-Dickinson).

#### Exercise and Beta Blocker Protocol

Participants completed the same maximal exercise test to determine 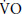
_2max_ (40.3 ± 10.2 mL/kg/minute), as stated above, but were required to visit the laboratory again on four separate occasions with a period of 7 days interspersed between visits (10). Participants performed the same 30-minute steady state or 20-minute graded bout of cycling exercise on three separate occasions after ingesting either (i) 10 mg bisoprolol (β1-AR antagonist), (ii) 80 mg nadolol (nonselective β1+β2-AR antagonist), or (iii) a placebo, exactly 3 hours prior to exercise (6). Blood samples were collected prior to drug/placebo ingestion (REST) and during the last 3–5 minutes of the EX. Heart rate, ECG activity, and respiratory gas exchange were recoded continuously throughout all trials. The tablets were administered in a double-blind fashion and the trials were performed using a block randomization design.

#### ISO Infusion Protocol

Participants were required to visit the Clinical and Translational Sciences Research Center (CATS) at The University of Arizona to complete the nonselective β-AR agonist infusion trial. Prior to infusion, two indwelling catheters (Becton-Dickinson) were placed inside bilateral antecubital veins to allow for simultaneous infusion of ISO and blood collection. Participants were infused with ISO (50 ng/kg/minute) for 20 minutes and blood samples were collected before (REST) and during the last 10 minutes of ISO infusion. Heart rate, ECG activity, and blood pressure were monitored during the trial.

### Blood Sample Analysis and Processing

Complete blood counts were immediately performed on whole blood samples treated with EDTA using an automated hematology analyzer (AcT 5Diff CP, Beckman Coulter). The enumeration of leukocytes and leukocyte subtypes in whole blood was performed using direct immunofluorescence assays and up to 8-color flow cytometry (MACSQuant 10, Miltenyi Biotec). All antibodies were purchased from Miltenyi Biotec unless otherwise stated. Briefly, peripheral blood mononuclear cells (PBMC) were stained with the following antibodies; CD8-VioBlue, CD14-VioGreen, CD3-FITC, CD4-PE, CD20-PerCP-Vio700, CD45-APC, CD56-APC-Vio770. Blood samples treated with ACD were used to isolate PBMCs (Ficoll-Paque PLUS, Cytiva), which were cryopreserved in liquid nitrogen at a concentration of 10 × 10^6^ cells/mL in freezing media (90% FBS, 10% DMSO) until they were used to phenotype and expand γδ T-cell *ex vivo*. Briefly, PBMCs were stained with the following antibodies; CD8-VioBlue, CD16-VioBlue, CD56-VioBlue, TRAIL-BV421 (BD Biosciences), CCR5-BV421 (BioLegend), CD3-VioGreen, Vδ2-FITC, CD4-PE, CD62L-PE, NKG2D-PE, CXCR3-PE, CD45-PerCP-Vio700, PD1-PerCP-eflour (BD Biosciences), 2B4-PerCP-Cy5.5 (BioLegend), BTLA-PerCP-Cy5.5 (BioLegend), TCRαβ-PE-Vio770, CD45RA-PE-Vio770, FasL-PE-Vio770, NKG2C-PE-Vio770, CCR7-PE-Vio770, Vδ1-APC, DNAM1-APC, NKG2A-APC, CD161-APC, TCRγδ-APC-Vio770, Vδ1-APC-Vio770. Extra aliquots were frozen from 3 participants for subsequent RNA isolation and single-cell RNA sequencing (scRNA-seq) using the 10x genomics platform.

### Expansion of γδ T-cells

γδ T-cells were expanded using methods described previously (10, 34). Briefly, PBMCs were thawed, enumerated, and seeded at a concentration of 1 × 10^6^ cells/mL in a 24 well-plate with culture media (RPMI1640 media +10% FBS and 10% penicillin-streptomycin) containing 300 IU/mL of IL2 (Miltenyi Biotec) and 5 µmol/L of ZOL (Sigma-Aldrich). Culture media was changed on days 3, 7, and 10, with fresh culture media culture containing IL2 (300 IU/mL) only. After 14 days, expanded Vγ9Vδ2+ T-cells were harvested to determine number, phenotype, and function by flow cytometry (MACSQuant 10; Miltenyi Biotec). Expanded Vγ9Vδ2+ T-cell phenotypes were assessed in a similar manner as previously described in the aforementioned section but with a slightly altered phenotype panel by replacing, Vδ1-APC-Vio770 with TCRγδ-APC-Vio770 and NKG2C-PE-Vio770 with FasL-PE-Vio770. The expanded Vγ9Vδ2+ T-cells were cryopreserved in liquid nitrogen at a concentration of 10 × 10^6^ cells/mL in freezing media (90% FBS, 10% DMSO) until use in the *in vitro* cytotoxicity assays, adoptive transfer experiments in xenogeneic mice, and bulk RNA-seq analysis.

### Vγ9Vδ2+ T-cell Cytotoxicity Assays

The *in vitro* cytotoxic function of the EX and ISO expanded Vγ9Vδ2+ T-cells were tested against the chronic myeloid leukemia cell line K562 (ATCC: CCL-243), multiple myeloma cell line U266 (ATCC: TIB-196), and Burkitt lymphoma cell line Daudi (ATCC: CCL-213). All target cells were maintained in a glutamine-enriched RPMI1640 media (+10% FBS and 10% penicillin-streptomycin). All experiments were performed with target cells between passages 3 and 9, to ensure all target cells used in this study were equivalent. *Mycoplasma* testing was routinely performed but we did not authenticate the cell lines. In some experiments, the target cells were incubated with 5 µmol/L of ZOL for 20 hours. On the day of the Vγ9Vδ2+ T-cell cytotoxicity assay, 2 × 10^6^ target cells were removed and labeled with an anti-CD71-FTIC antibody. The target cells were washed and resuspended in 5 mL of RPMI1640 media (+10% FBS and 10% penicillin-streptomycin). The expanded Vγ9Vδ2+ T-cells were then cocultured with the CD71-labeled target cells in a 96-well plate at 0:1, 1:1, 5:1. 10:1, and 20:1 γδ T-cell:target cells (E:T) ratios in a final volume of 200 µL. The 0:1 E:T ratio was used to determine the spontaneous death of the target cells. After 4 hours of incubation at 37°C, the cytotoxicity of γδ T-cell was assessed on the MACSQuant 10 (Miltenyi Biotec). Propidium iodide was added to each well on the 96-well plate immediately before analysis to quantify cell death. Vγ9Vδ2+ T-cell cytotoxic activity was quantified as specific lysis (% total lysis − % spontaneous death).

The receptor-ligand mechanism by which expanded Vγ9Vδ2+ T-cell recognize and lyse K562 cells was determined using antibody blocking experiments. To block activation of Vγ9Vδ2+ T-cells through the NKG2D, TRAIL, DNAM-1, and TCRγδ receptors, expanded Vγ9Vδ2+ T-cells were incubated with either media alone, an irrelevant antibody, or anti-mAb against the activating receptors for 30 minutes prior to performing the Vγ9Vδ2+ T-cell cytotoxicity assay. Concanamycin A (CMA) was used to inhibit the perforin-mediated killing of target cells by Vγ9Vδ2+ T-cells. CMA was incubated with Vγ9Vδ2+ T-cells at a concentration of 15 nm for 30 minutes at 37°C prior to coculture with target cells, without further washing. To block recognition of tumor cells through the PVR, Nectin-2, TRAIL-R1, and TRAIL-R2, K562 cells were incubated with either media alone, an irrelevant antibody, or anti-mAb against the respective tumor ligands for 30 minutes at 37°C prior to performing the Vγ9Vδ2+ T-cell cytotoxicity assay.

### Xenogeneic Mouse Experiments

NOD.Cg-Prkdc^scid^ Il2rg^tm1Wjl^ Tg(IL15)1Sz/SzJ [NSG-Tg(Hu-IL15)] mice (Jackson Labs, stock no: 030890; RRID:MGI:6201748) between the ages of 8–12 weeks were used for xenotransplantation of expanded Vγ9Vδ2+ T-cells and K562-luc2 tumor cells (ATCC: CCL-243-LUC2). All experiments were performed with K562-luc2 between passages 3 and 4, to ensure all target cells used in this study were equivalent. *Mycoplasma* testing was routinely performed but we did not authenticate the cell line. K562-luc2 cells were maintained at 37°C, 5% CO_2_ in Iscove's DMEM supplemented with 10% FBS and 8 µg/mL blasticidin and prepared for tail vein injection as described previously (35). Mice were irradiated with 100 cGy, using a Cesium 137 irradiator, one day (day −2) before intravenous injection of expanded Vγ9Vδ2+ T-cells on day −1, mice not receiving expanded γδ T-cells (tumor-only) were injected with an equal volume of saline on the same day. The following day (day 0), 1 × 10^6^ K562-luc2 cells were injected intravenously through the lateral tail vein. One day after tumor injection (day 1), mice will be exposed to bioluminescent imaging (BLI), to monitor tumor progression using a LagoX (Spectral Instruments Imaging). Mice were monitored daily until sacrifice. In some experiments, ZOL was injected intraperitoneal on day 1 and repeated weekly to sensitize the tumors to γδ T cell–mediated lysis. BLI was repeated every 3–4 days to track tumor progression. Briefly, d-luciferin, potassium salt (15 mg/mL; Gold Biotechnologies) was injected intraperitoneally at a concentration of 10 µL/g of body weight (BW). Data were expressed as photons/second to allow for comparisons between exposure times. Although expanded γδ T-cells do not elicit GvHD, symptoms were monitored to ensure the small proportion of contaminating αβ T-cells (∼5%–10%) did not propagate GvHD. Mice were weighed and scored every 3–4 days to monitor GvHD. The following symptoms were assessed to determine GvHD status: Skin Integrity (0–2): 0 = normal, healthy skin; 1 = Scaling of paws/tail; 2 = dehydrated, obvious areas of denuded skin; Fur Integrity (0–2): 0 = normal, fluffy, and elastic fur; 1 = mild to moderate ruffling; 2 = soiled, stiff, and rough fur; Posture (0–2): 0 = normal posture; 1 = hunching only at rest; 2 = severe hunching, sunken or distended abdomen; Activity (0–2): 0 = normal, responsive and vocal; 1 = mild to moderately decreased; 2 = unresponsive, separates from group, circling, head pressing; Weight Loss (0–2): 0 ≤ 10%; 1 = 10% to 20%; 2 ≥ 20%; Diarrhea (0–1): 0 = no; 1 = yes. All animal procedures were performed in accordance with protocols approved by the University of Arizona Institutional Animal Care and Use Committee (#17-338).

### RNA-seq Analysis

We used scRNA-seq (*n* = 3) to identify transcriptomic changes within exercise-mobilized Vγ9Vδ2+ T-cells, and bulk RNA-seq to identify transcriptomic shifts among Vγ9Vδ2+ T-cells expanded, from the same participants, after both exercise (*n* = 2) and ISO infusion (*n* = 2). PBMCs or expanded Vγ9Vδ2+ T-cells (>95% purity) were thawed and resuspended in PBS and RNAlater solution and processed by the University of Arizona Genetics Core for scRNA-seq analysis using the 10x Genomics platform and bulk RNA-seq using the Illumina NextSeq500 platform. The scRNA-seq library generation, sequencing, and analysis were performed as described previously (35, 36). For bulk RNA-seq, RNA samples’ quality was assessed with a high sensitivity RNA Fragment Analyzer Kit (Advanced Analytics) and quantity with an RNA HS assay kit (Qubit). Quality samples were used for library builds with the Rapid RNA Library Kit (Swift) and Dual Combinatorial Indexing Kit (Swift). Samples had quality and average fragment size assessed with the High Sensitivity next-generation sequencing (NGS) Analysis Kit (Advanced Analytics). Quantity was assessed with the Kapa Library Quantification kit (Illumina), and then samples were equimolar-pooled and clustered for sequencing using Illumina NextSeq500 run chemistry (NextSeq 500/550 High Output v2 kit 150 cycles). Data were analyzed by the Bioinformatics core. For each differential expression analysis comparison, gene set enrichment analysis (GSEA), with a FDR of (0.25), was performed and annotated to both Kyoto Encyclopedia of Genes and Genomes (KEGG) and Gene Ontology (GO) terms.

### Statistical Analysis

Statistical analyses were completed using GraphPad Prism 9 (GraphPad Software) or SPSS (v24.0 IBM). All data are represented as mean ± SD unless otherwise stated. Paired *t* tests or Wilcoxon matched-pair signed rank test were used to detect differences in the proportion, total number, and phenotypes of isolated γδ T-cells or expanded Vγ9Vδ2+ T-cells before and during exercise or ISO infusion. Multiple linear mixed models (LMM) with Bonferroni correction for multiple comparisons were built to detect main effects of group (REST vs. EX/ISO), dose (E:T ratios), condition (antibody blockade), treatment (ZOL sensitization), and multiple interaction effects for *in vitro* cytotoxic function of the EX and ISO expanded Vγ9Vδ2+ T-cells. Multiple LMMs with Bonferroni correction for multiple comparisons were used to detect main effects of group, time, treatment, and multiple interaction effects for leukemic burden (BLI) and BW. Simple OS (Kaplan–Meier) was used to detect differences in OS and tumor-free survival (TFS). Significance was accepted at *P* < 0.05. For GSEA, the FDR was used separately for each database (GO and KEGG) to correct for multiple hypothesis testing and given the exploratory nature of our analysis, we selected an FDR threshold of <0.25, which denotes the confidence of “possible” or “hypothesis,” while an FDR <0.05 denotes “high confidence” or “statistical significance” (13). We used the less stringent FDR for our GSEA to avoid overlooking potentially meaningful changes in enriched gene sets in response to exercise.

### Data Availability

The scRNA-seq and bulk RNA-seq data generated in this study are publicly available in NCBI's Gene Expression Omnibus at accession numbers GSE212740 and GSE263181, respectively. All other data generated during this study are available from the corresponding author upon request.

## Results

### Acute Exercise Mobilizes γδ T-cells with a Surface Phenotype and Transcriptomic Profile Associated with Increased Effector Function

**FIGURE 1:**
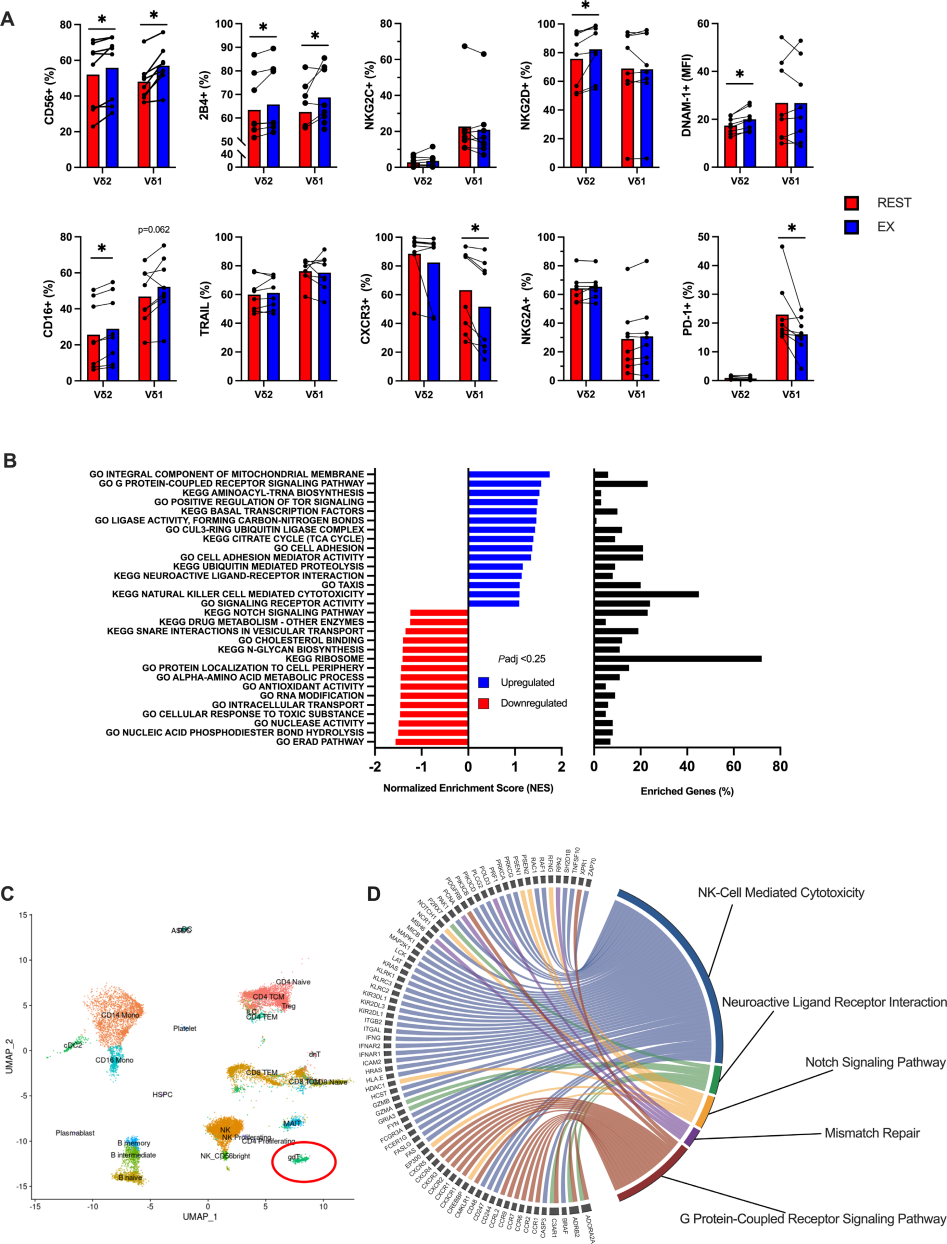
Exercise preferentially mobilizes γδ T-cells with phenotypic and transcriptomic profiles associated with anti-tumor immunity. **A,** The percentage or MFI of activating, inhibitory, and chemokine receptors among Vδ2+ and Vδ1+ T-cells before (REST) and during (EX) acute exercise (*n* = 8). Significance is indicated by * (*P* < 0.05). **B,** GSEA performed using KEGG and GO terms showing enriched pathways on exercise mobilized γδ T-cells (*n* = 3). Graphs show enriched upregulated (blue) and downregulated (red) pathways as well as the percentage of DEGs contributing to each mechanism. Statistical significance set as FDR ≤ 0.25. **C,** Azimuth map for human PBMCs showing the 26 clusters identified by scRNA-seq at rest; γδ T-cells mapping is circled in red. (*n* = 3). **D,** Chord diagram displaying the leading-edge genes driving the enrichment of terms associated with cytotoxicity and anti-tumor signaling in γδ T-cells (*n* = 3). Data are represented as mean; *, *P* < 0.05, Students two-tailed paired *t* test (A); * ≤0.25, FDR (B).

A single bout of cycling exercise mobilized lymphocytes to the blood compartment, increasing total numbers of all major lymphocyte subtypes (CD3^+^ T-cells, CD4^+^ T-cells, CD8^+^ T-cells, NK-cells, γδ T-cells; refs. 10, 37–39). Both Vδ2+ and Vδ1+ T-cells made up a larger proportion of CD3^+^ T-cells during the EX (Table 1). Our flow cytometric panel was designed to detect exercise-induced changes among circulating Vδ2+ and Vδ1+ T-cells for markers associated with activation, inhibition, homing, and exhaustion. Exercise increased the percentage of Vδ2+ and Vδ1+ T-cells expressing CD56 and 2B4 (Fig. 1A). While the percentage of NKG2D+ and CD16+ and mean fluorescence intensity (MFI) of DNAM-1, CXCR3, and CCR5 were also elevated among the exercise-mobilized Vδ2+ T-cells (Fig. 1A; Supplementary Fig. S1). Exercise reduced the percentage of PD-1+, CXCR3+, and CCR7+ cells among the Vδ1+, while the MFI of NKG2A was decreased among the Vδ2+ T-cells (Fig. 1A; Supplementary Fig. S1). Vδ1+ and Vδ2+ T-cell expression of NKG2C, TRAIL, Fas Ligand (FasL), CD272, and CD161 was unaffected by exercise (Fig. 1A; Supplementary Fig. S1). To understand if exercise altered gene transcription among γδ T-cells in blood, we identified pan γδ T-cells by scRNA-seq using the Azimuth map for human PBMCs before quantifying differentially expressed genes (DEG) and performing GSEA on the γδ T-cell cluster, which we annotated to GO and KEGG terms (*n* = 3; Fig. 1B and C). The top 15 pathways upregulated and downregulated in response to exercise are shown. Notable enriched gene sets among γδ T-cells mobilized by exercise include “G protein–coupled receptor signaling,” “citrate cycle (tricarboxylic cycle),” “cell adhesion,” “neuroactive ligand–receptor interaction,” “chemotaxis,” and “NK cell–mediated cytotoxicity” (Fig. 1D). Collectively, these findings indicate that exercise mobilizes γδ T-cells with a surface protein phenotype and transcriptomic profile associated with increased effector functions, including those related to cytotoxicity, migration, activation, and signaling.

**TABLE 1:** The effect of acute exercise on lymphocyte subsets

	REST	EX	*t* or *z* score	*P*-value
Lymphocytes				
cells/µL	1,586 ± 464	3,233 ± 1,112****	*t* = 7.327	<0.0001
CD3^+^ T-cells				
cells/µL	1,204 ± 385	1,877 ± 642*	*t* = 6.319	0.004
of Lymphocytes (%)	72.9 ± 5.2	57 ± 5.7****	*t* = 11.11	<0.0001
CD4^+^ T-cells				
cells/µL	745 ± 220	955 ± 296*	*t* = 5.162	0.0013
of Lymphocytes (%)	45.4 ± 5	30.8 ± 5.4*	*t* = 14.72	<0.0001
of CD3^+^ T-cells (%)	62.3 ± 4.9	53.9 ± 7.2***	*t* = 7.275	0.0010
CD8^+^ T-cells				
cells/µL	369 ± 126	673 ± 275*	*t* = 5.312	0.0011
of Lymphocytes (%)	22.2 ± 2.8	20 ± 3*	*t* = 2.476	0.0425
of CD3^+^ T-cells (%)	30.5 ± 3.4	35.2 ± 5**	*t* = 4.658	0.0020
γδ T-cells				
cells/µL	86 ± 77	225 ± 183**	*t* = 3.534	0.0095
of Lymphocytes (%)	4.9 ± 3	6.7 ± 3.7**	*t* = 3.654	0.0081
of CD3^+^ T-cells (%)	6.8 ± 4	11 ± 6.3**	*t* = 3.955	0.0055
Vδ2+ T-cells				
cells/µL	76 ± 74	181 ± 177*	*t* = 2.851	0.0247
of Lymphocytes (%)	4.3 ± 3.1	5.4 ± 3.9*	*t* = 2.796	0.0267
of CD3^+^ T-cells (%)	6 ± 4.2	9.1 ± 6.7*	*t* = 3.125	0.0167
Vδ1+ T-cells				
cells/µL	5 ± 8	29 ± 62**	*z* = 2.521	0.0120
of Lymphocytes (%)	0.3 ± 0.6	0.8 ± 1.7*	*z* = 2.100	0.0360
of CD3^+^ T-cells (%)	0.6 ± 0.7	1.9 ± 2.5*	*z* = 2.521	0.0120
NK-Cells				
cells/µL	188 ± 79	928 ± 426***	*t* = 5.797	0.0007
of Lymphocytes (%)	11.1 ± 3.2	26.6 ± 6****	*t* = 11.14	<0.0001

NOTE: The absolute number and percentage of lymphocytes and lymphocyte subsets before (REST) and during exercise (EX). Depending on the statistical test used a t-score or z-score is reported, respectively. Data are mean ± SD (*n* = 8). Statistical differences from REST are indicated by *, *P* > 0.05; **, *P* > 0.01; ***, *P* > 0.001; ****, *P* > 0.0001.

### Acute Exercise Augments the *Ex Vivo* Expansion and Antileukemic Activity of Vγ9Vδ2+ T-cells *In Vitro* and *In Vivo*

**FIGURE 2:**
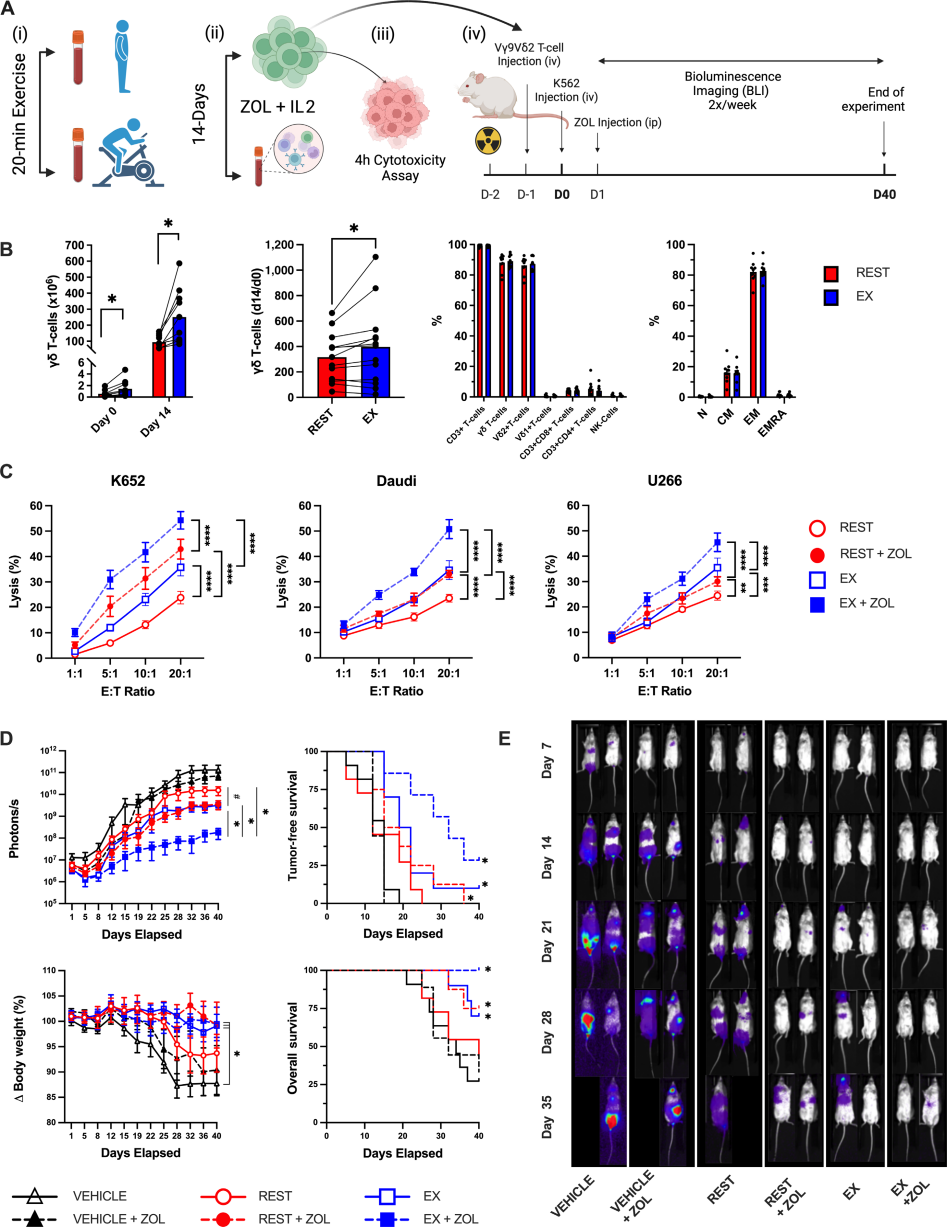
Acute exercise enhances the antileukemic activity of *ex vivo* expanded Vγ9Vδ2+ T-cells *in vitro* and *in vivo*. **A,** Schematic of the experimental design for *in vitro* and *in vivo* experiments. **B,** The total number (1 × 10^6^) of Vγ9Vδ2+ cells isolated before (REST) and during the final 5 minutes of exercise (EX) at day 0, and the total number of Vγ9Vδ2+ T-cells generated in the expanded cell products after stimulation with ZOL+IL2 for 14 days. The number of Vγ9Vδ2+ cells generated at day 14 relative to the number of γδ cells in the PBMC fractions at day 0. The cellular composition of the expanded Vγ9Vδ2+ T-cell products at day 14. The proportions of naïve (N), CM, EM, and CD45RA+ EMRA cells among Vγ9Vδ2+ T-cells after 14 days of expansion (*n* = 10). **C,** The *in vitro* anti-tumor activity of *ex vivo* expanded Vγ9Vδ2+ T-cells against K562, Daudi, and U266 with and without ZOL sensitization. Target cells were exposed to 5 µmol/L of ZOL for 20 hours and cytotoxicity of Vγ9Vδ2+ cells was assessed via flow cytometry–based assays. **D,** To determine the *in vivo* GvL effect of exercise expanded Vγ9Vδ2+, NSG-IL15 mice were injected with REST or EX expanded Vγ9Vδ2+ (10 × 10^6^) and challenged with 1 × 10^6^ luciferase tagged human chronic myeloid leukemia cells (K562-luc). A subset of mice in each group were also provided weekly injections of ZOL (REST+ZOL; EX+ZOL; VEHICLE +ZOL). The BLI (photons/second) scores, TFS, change in BW, and overall probability of survival (OS) after injection of expanded Vγ9Vδ2+ T-cells with or without ZOL sensitization (*n* = 8–11/group**)**. BLI significant difference from EX+ZOL and EX conditions indicated by * and ^#^, respectively (all conditions were significantly different from vehicle controls). TFS, BW, and OS significant difference from vehicle controls were indicated by *. **E,** Representative bioluminescence images of leukemia-bearing mice that received (from left to right) vehicle, vehicle + ZOL, REST expanded Vγ9Vδ2+ T-cells, REST expanded Vγ9Vδ2+ T-cells + ZOL, EX expanded Vγ9Vδ2+ T-cells, and EX expanded Vγ9Vδ2+ T-cells + ZOL. BLI intensity on a scale from low (purple) to high (red). Data are represented as mean ± SEM; *, *P* < 0.05; **, *P* < 0.01; ***, *P* < 0.001; ****, *P* < 0.0001 by Student two-tailed paired *t* test (B); repeated measures two-way ANOVA or LMM with Bonferroni *post hoc* test (C–D); log-rank (Mantel–Cox) test (D).

We showed previously that exercise augmented the *ex vivo* expansion of Vγ9Vδ2+ T-cells and increased their ability to kill K562, U266, and 221.AEH target cells *in vitro* (10). Here, we added an additional lymphoma cell line (Daudi) and determined whether ZOL sensitization would enhance the antileukemic effects of the Vγ9Vδ2+ T-cells expanded after exercise (Fig. 2A). Exercise increased the total number of Vγ9Vδ2+ T-cells expanded over 14 days, even after adjusting for Vγ9Vδ2+ T-cell numbers among the stimulated PBMC fractions at day 0 (Fig. 2B). The purity (∼90% γδ T-cells) and subset [naïve, central memory (CM), effector memory (EM), effector memory RA (EMRA)] composition of the expanded products was not altered by exercise (Fig. 2B). However, the Vγ9Vδ2+ T-cells expanded after exercise have significantly enhanced cytotoxicity against K562, Daudi and U266 target cells *in vitro* both with and without ZOL sensitization, indicating that Vγ9Vδ2+ T-cells expanded from exercise-mobilized cells have enhanced anti-tumor activity against multiple hematologic cancer cell lines (Fig. 2C). To determine whether Vγ9Vδ2+ T-cells expanded after exercise would persist and control leukemic growth *in vivo*, we engrafted irradiated NSG(Tg)-IL15 mice with a luciferase tagged K562 cells (1 × 10^6^), and, the following day, injected a vehicle control (saline) or Vγ9Vδ2+ T-cells (10 × 10^6^) expanded from resting or exercise-mobilized PBMCs (Fig. 2A). Half of the mice in each group received weekly injections of ZOL starting on day+ 2 and all mice were monitored for up to 40 days. Mice were removed if their BW dropped by 20% or more, or if they presented with a morbidity score ≥8. BLI performed 2x/week revealed significantly lower tumor burden (photons/second) in mice receiving Vγ9Vδ2+ T-cells expanded after exercise when combined with ZOL (Fig. 2D and E). TFS (BLI score > baseline) and OS was significantly greater in mice receiving Vγ9Vδ2+ T-cells expanded after exercise with ZOL compared with all other groups (Fig. 2D). Collectively, these findings indicate that exercise mobilizes Vγ9Vδ2+ T-cells to blood which when expanded *ex vivo* have enhanced anti-tumor activity both *in vitro* and *in vivo*.

### β2 but not β1-AR Activation is Responsible for Mobilizing Vγ9Vδ2+ T-cells to Blood and Augmenting Their Cytotoxic Activity Following *Ex Vivo* Expansion

**FIGURE 3:**
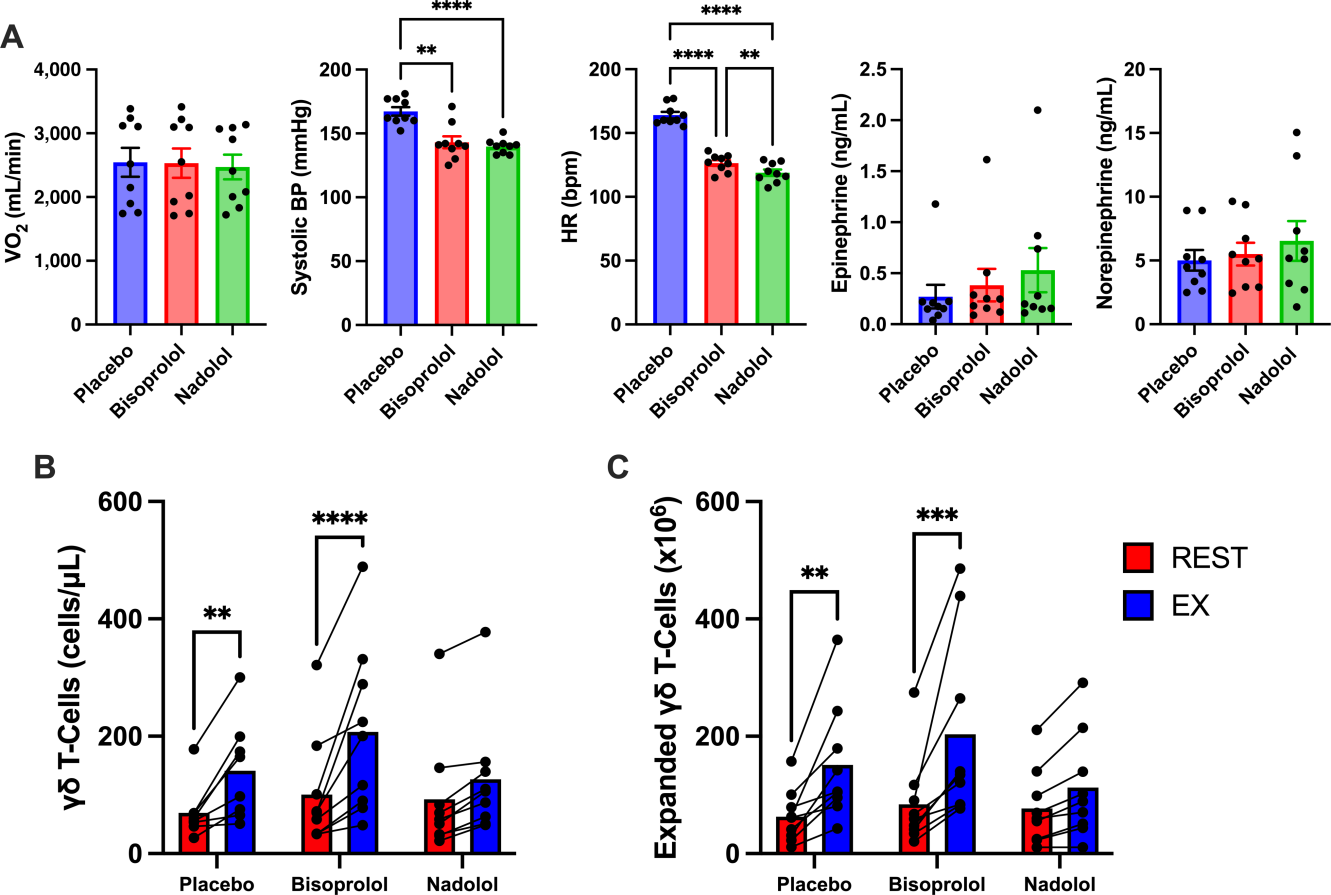
Blockade of β_2_-AR blunts the mobilization and expansion of Vγ9Vδ2+ T-cells. **A,** The physiologic effects of a placebo, β1-AR antagonist (10 mg bisoprolol), or a nonselective β1+β2 antagonist (80 mg, nadolol) on VO_2_ (mL/minute), systolic blood pressure (mmHg), HR (bpm), epinephrine (ng/mL), and norepinephrine (ng/mL) during the last minutes of acute exercise (*n* = 9). The effects of a placebo, β1-AR antagonist, or a nonselective β1+β2 antagonist on the total number of Vγ9Vδ2+ T-cells mobilized with exercise (**B**) and the *ex vivo* expansion of Vγ9Vδ2+ T-cells after 14 days with ZOL+IL2 (*n* = 9; **C**). Data are represented as mean ± SEM; *, *P* < 0.05; **, *P* < 0.01; ***, *P* < 0.001; ****, *P* < 0.0001 by repeated measures one-way ANOVA with Bonferroni *post hoc* test.

**FIGURE 4:**
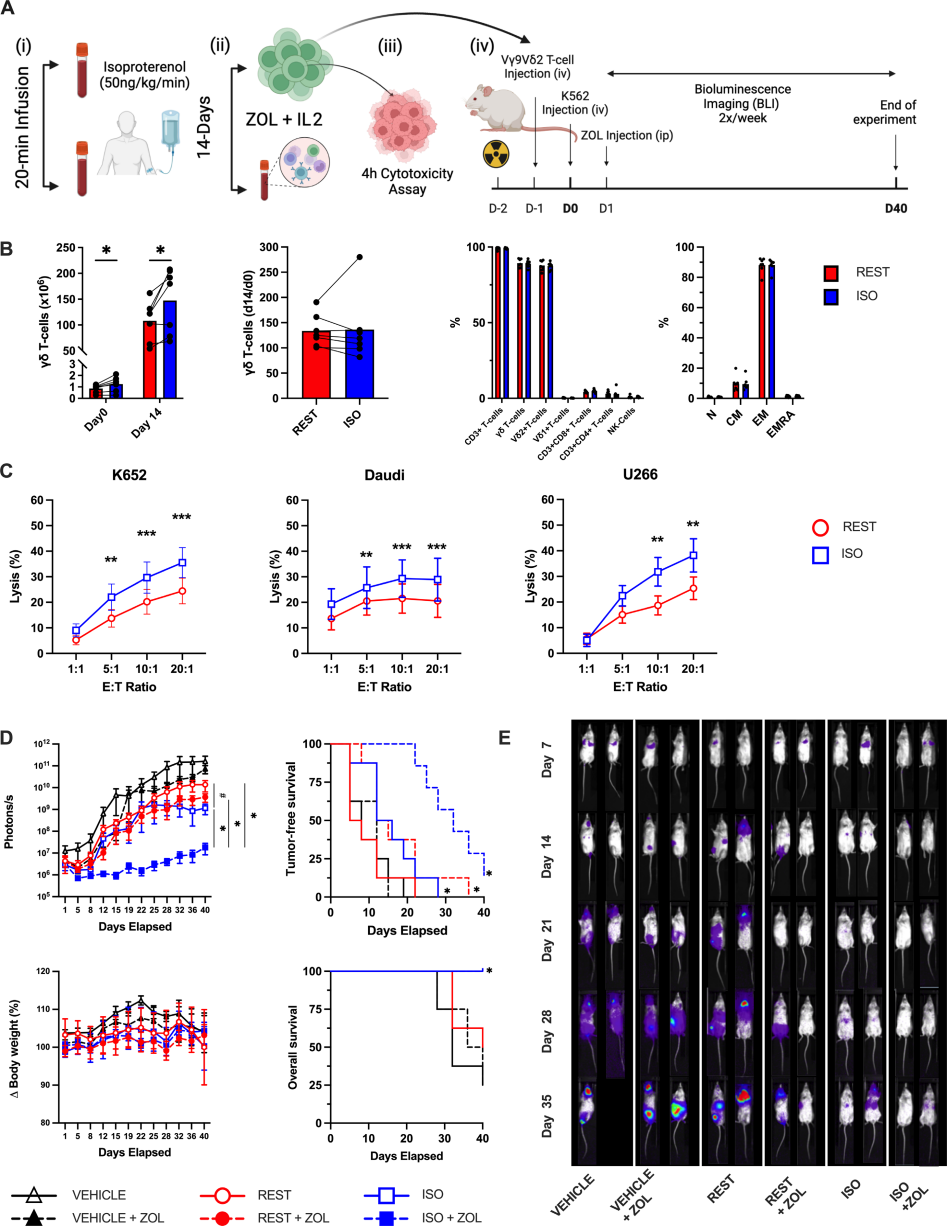
ISO infusion enhances the antileukemic activity of *ex vivo expanded* Vγ9Vδ2+ T-cells *in vitro* and *in vivo*. **A,** Schematic of the experimental design for *in vitro* and *in vivo* experiments. **B,** The total number (1 × 10^6^) of Vγ9Vδ2+ cells isolated before (REST) and during the final 5 minutes of ISO infusion at day 0, and the total number of Vγ9Vδ2+ T-cells generated in the expanded cell products after stimulation with ZOL+IL2 for 14 days. The number of Vγ9Vδ2+ cells generated at day 14 relative to the number of γδ cells in the PBMC fractions at day 0. The cellular composition of the expanded Vγ9Vδ2+ T-cell products at day 14. The proportions of naïve (N), CM, EM, and CD45RA+ EMRA cells among Vγ9Vδ2+ T-cells after 14 days of expansion (*n* = 7). **C,** The *in vitro* anti-tumor activity of *ex vivo* expanded Vγ9Vδ2+ T-cells against K562, Daudi, and U266 cells via flow cytometry–based assays. Significance indicated by ** (*P* < 0.01) and *** (*P* < 0.001). **D,** To determine the *in vivo* GvL effect of ISO expanded Vγ9Vδ2+, NSG-IL15 mice were injected with REST or ISO expanded Vγ9Vδ2+ (10 × 10^6^) and challenged with 1 × 10^6^ luciferase tagged human chronic myeloid leukemia cells (K562-luc). A subset of mice in each group were also provided weekly injections of ZOL (REST+ZOL; ISO+ZOL; VEHICLE +ZOL). The BLI (photons/second) scores, TFS, change in BW, and probability of survival after injection of expanded Vγ9Vδ2+ T-cells with or without ZOL sensitization (*n* = 7–8/group**).** BLI significant difference from EX+ZOL and EX conditions indicated by * and ^#^, respectively (all conditions were significantly different from vehicle controls). TFS, BW, and OS significant difference from vehicle controls were indicated by * (TFS; EX, EX+ZOL, and REST+ZOL significance indicated by a single marker). **E,** Representative bioluminescence images of leukemia-bearing mice that received (from left to right) vehicle, vehicle + ZOL, REST expanded Vγ9Vδ2+ T-cells, REST expanded Vγ9Vδ2+ T-cells + ZOL, ISO expanded Vγ9Vδ2+ T-cells, and ISO expanded Vγ9Vδ2+ T-cells + ZOL. BLI intensity on a scale from low (purple) to high (red). Data are represented as mean ± SEM; *, *P* < 0.05; **, *P* < 0.01; ***, *P* < 0.001; ****, *P* < 0.0001 by Students two-tailed paired *t* test (B); Repeated measures two-way ANOVA or LMM with Bonferroni *post hoc* test (C–D); log-rank (Mantel–Cox) test (D).

To determine the role of β1- and β2-AR signaling on the exercise effects, we first administered a placebo, a selective β1-AR antagonist (10 mg bisoprolol), or a nonselective β1+β2-AR antagonist (80 mg, nadolol) to healthy participants 3 hours prior to exercise. The three exercise trials were performed in a double-blind randomized cross-over design with each subject acting as his/her own control. A 7-day wash out period was interspersed between each 20-minute exercise trial and blood was collected before and during the last 5 minutes of exercise. Absolute cycling power (watts) was controlled across all three exercise trials. Bisoprolol and nadolol did not affect exercising oxygen uptake or plasma catecholamines (epinephrine and norepinephrine) but did lower exercising heart rate and systolic blood pressure by a similar extent compared with placebo (Fig. 3A). This indicates that hemodynamic forces (e.g., shear stress), which can also mobilize lymphocytes to the circulation, was similar between the bisoprolol and nadolol trials (6). Nadolol but not bisoprolol significantly blunted both the mobilization and *ex vivo* expansion of Vγ9Vδ2+ T-cells in response to exercise, revealing that the exercise effects are dependent on β2-AR but not β1-AR activation (Fig. 3B and C). We next aimed to determine whether systemic β-AR activation in the absence of exercise could also mobilize and increase the function of expanded Vγ9Vδ2+ T-cells. To do this, we infused healthy participants (*n* = 7) with the synthetic β1+β2-AR agonist, ISO (50 ng/kg/minute) continuously for 20 minutes under resting conditions (participants remained supine during the infusion; Fig. 4A). Blood was collected before and during the last 5 minutes of infusion. Like exercise, ISO mobilized Vγ9Vδ2+ T-cells to blood and increased the total number of Vγ9Vδ2+ T-cell expanded with ZOL+IL2 over 14 days (Fig. 4B). Contrary to exercise, the numbers of Vγ9Vδ2+ T-cells expanded at day 14 was proportional to the numbers of Vγ9Vδ2+ T-cells in the PBMC fractions stimulated at day 0 (Fig. 4B). However, like exercise, Vγ9Vδ2+ T-cells expanded from cells collected during ISO infusion were still superior at killing K562, Daudi and U266 target cells *in vitro* (Fig. 4C). We then tested whether Vγ9Vδ2+ T-cells expanded after ISO infusion would also exert better control of leukemic growth *in vivo*. Using the same NSG(Tg)IL15 mouse model for the previously described exercise trials, we observed significantly lower tumor burden (photons/second) in mice receiving Vγ9Vδ2+ T-cells expanded after ISO infusion when combined with ZOL (Fig. 4D and E). Similar to exercise, TFS (BLI score > baseline) and OS was significantly greater in mice receiving Vγ9Vδ2+ T-cells expanded after ISO infusion with ZOL compared with all other groups (Fig. 4D). Collectively, these findings indicate that β2 but not β1-AR activation is required to mobilize Vγ9Vδ2+ T-cells to blood that can be expanded into a superior product with enhanced cytotoxic activity against human hematologic malignancies.

### Exercise and ISO Infusion Evoke Similar Surface Phenotype and Transcriptomic Shifts Among *Ex Vivo* Expanded Vγ9Vδ2+ T-cells

**FIGURE 5:**
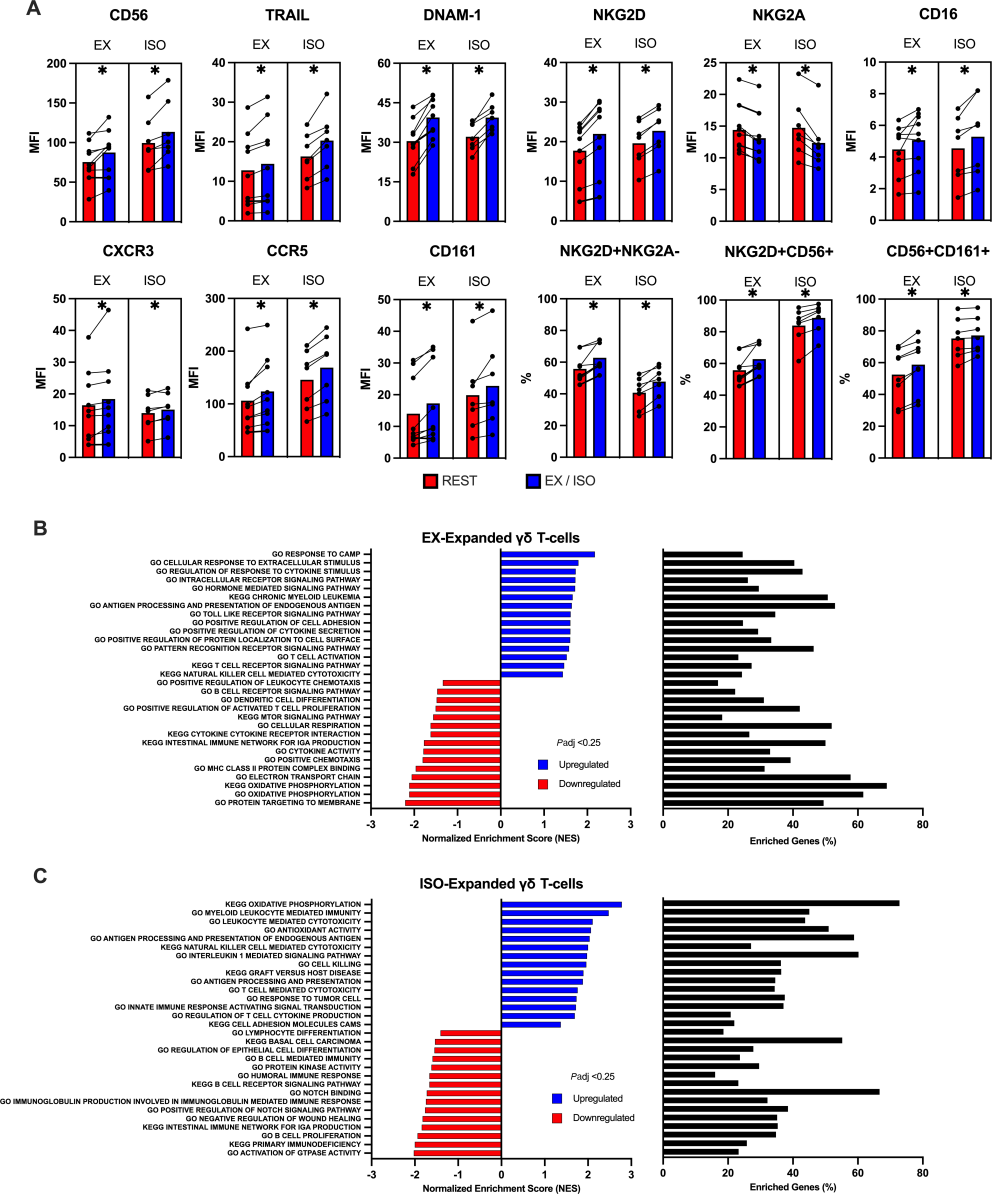
Systemic β-AR activation through either acute exercise or ISO infusion evoke similar transcriptomic and surface phenotype shifts among *ex vivo* expanded Vγ9Vδ2+ T-cells. **A,** The percentage of activating, inhibitory, and chemokine receptors among expanded Vγ9Vδ2+ T-cells before (REST) and during (EX) exercise or ISO infusion. (*n* = 8). Statistical significance set as *, *P* < 0.05. GSEA performed using KEGG and GO terms showing enriched pathways on expanded Vγ9Vδ2+ T-cells that were expanded after (**B**) exercise or (**C**) ISO infusion. Graphs show enriched upregulated (blue) and downregulated (red) pathways as well as the percentage of DEGs contributing to each mechanism. Statistical significance set as FDR ≤ 0.25. Data are represented as mean.

To identify potential phenotypic determinants of the exercise/ISO effects, we performed extended flow cytometry and bulk RNA-seq of the Vγ9Vδ2+ T-cell products expanded after exercise and ISO infusion. Pairwise comparisons were made with Vγ9Vδ2+ T-cells expanded from resting blood samples. Shifts in the surface phenotypes of Vγ9Vδ2+ T-cell expanded after exercise were strikingly similar to those expanded after ISO infusion (Fig. 5A). Surface expression of markers associated with cytotoxicity and migration such as CD56, TRAIL, DNAM-1, NKG2D, CXCR3, CCR5, and CD161 were significantly elevated among Vγ9Vδ2+ T-cells expanded after both exercise and ISO infusion (Fig. 5A). Conversely, NKG2A, a potent inhibitory receptor expressed by NK-cells and Vγ9Vδ2+ T-cells, was significantly lower after both exercise and ISO infusion (Fig. 5A). Bulk RNA-seq revealed several enriched gene sets among Vγ9Vδ2+ T-cells expanded after exercise including those related to “NK-cell mediated cytotoxicity,” “cell adhesion,” “T-cell activation,” “cAMP response,” and “antigen processing and presentation” (Fig. 5B). Among Vγ9Vδ2+ T-cells expanded after ISO infusion was an enrichment of gene sets related to “membrane protein targeting,” “oxidative metabolism,” “NK-cell mediated immunity,” “immune responses to tumor cell,” and “cytokine signaling” (Fig. 5C). Collectively, these findings indicate that exercise promotes the mobilization of Vγ9Vδ2+ T-cells that can be expanded into a cell product with a surface phenotype and transcriptomic profile associated with enhanced effector functions, and that the exercise effects can be replicated with systemic β2-AR activation *in vivo*.

### Exercise Arms Expanded Vγ9Vδ2+ T-cells with DNAM-1 to Increase Cytolysis of K562 via PVR and Nectin-2

**FIGURE 6:**
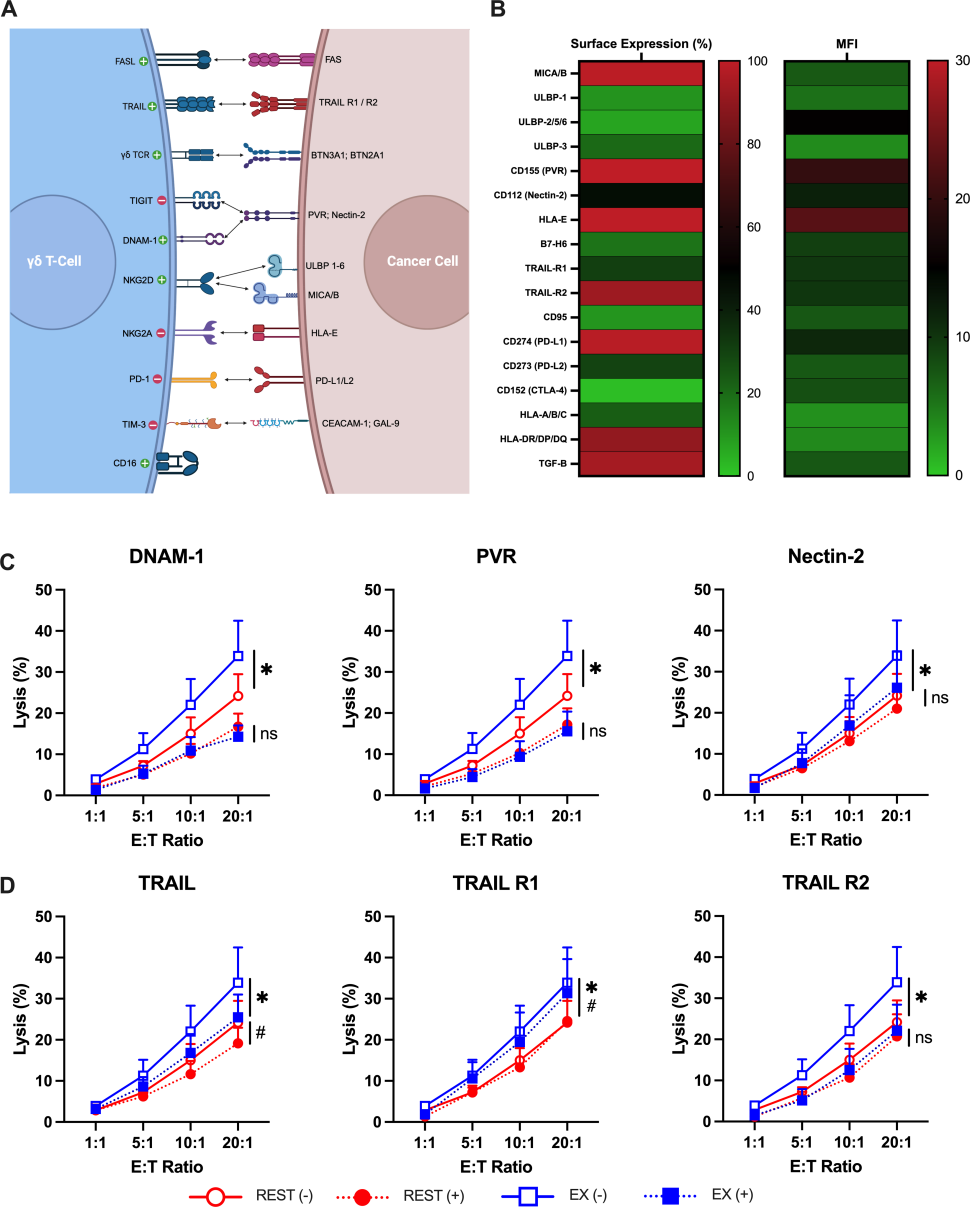
Interactions between DNAM-1 and PVR/Nectin-2 enhances the antileukemic activity of exercise expanded Vγ9Vδ2+ T-cells. **A,** Illustration of the receptors expressed on Vγ9Vδ2+ T-cells and their corresponding ligands expressed on cancer cells. **B,** Heat map of the surface expression and MFI of ligands and receptors on K562 cells. **C** and **D,** The specific lysis of K562 cells by expanded Vγ9Vδ2+ T-cells under two conditions: unstained control (−) and respective blocking antibody (+), DNAM-1, PVR, Nectin-2, TRAIL, TRAIL-R1, and TRAIL-R2 (*n* = 3). Significant differences between the between the unstained (−) and blocking conditions (+) were indicated by * and ^#^, respectively. Data are represented as mean ± SEM; *, *P* < 0.05; **, *P* < 0.01; ***, *P* < 0.001; ****, *P* < 0.0001 by repeated measures two-way ANOVA with Bonferroni *post hoc* test (C–D).

Our next step was to determine whether phenotypic shifts among the Vγ9Vδ2+ T-cells expanded after exercise were responsible for their enhanced cytotoxic activity (Fig. 6A). We showed previously that an upregulation of NKG2D on Vγ9Vδ2+ T-cells expanded after exercise played a mechanistic role in improved killing of the U266 multiple myeloma cell line, but not against the K562 chronic myeloid leukemia cell line (10). This was most likely due to K562-expressing lower levels of the NKG2D ligands ULBP-1 and ULBP-3 (Fig. 6B). We screened K562 cells for ligands of known activating and inhibitory receptors expressed by Vγ9Vδ2+ T-cells (Fig. 6B). We found that they expressed ligands for DNAM-1 in PVR and Nectin-2, and for TRAIL in TRAIL-R1 and TRAIL-R2 (Fig. 6B). We, therefore, identified DNAM-1 and/or TRAIL upregulation as a potential mechanism for the enhanced antileukemic effects of Vγ9Vδ2+ T-cells expanded after exercise. Vγ9Vδ2+ T-cells expanded after rest and exercise were cocultured with K562 cells with an anti-DNAM-1 antibody or isotype control for 4 hours (*n* = 3). DNAM-1 blockade not only lowered K562 killing, but also abrogated the enhanced cytotoxic effects seen with exercise-expanded Vγ9Vδ2+ T-cells (Fig. 6C). Furthermore, blocking the DNAM-1 ligands PVR and Nectin-2 expressed on the K562 cells similarly reduced K562 killing and abrogated the exercise effects (Fig. 6C). In a similar approach, blocking TRAIL was found to reduce K562 killing but the cytotoxic effects of exercise-expanded Vγ9Vδ2+ T-cells was still enhanced, indicating that the upregulation of TRAIL on Vγ9Vδ2+ T-cells expanded after exercise is not mechanistically involved in the enhanced cytotoxic effects against K562 (Fig. 6D). As expected, therefore, blocking TRAIL ligands (R1 and R2) on K562 cells did not alter the exercise effects (Fig. 6D). Antibody blocking the γδ-TCR and depleting perforin with CMA confirmed that the expanded Vγ9Vδ2+ T-cells exert their cytotoxic effects via activation of the γδ-TCR and release of cytolytic granules (Supplementary Fig. S2). These findings indicate that exercise upregulates DNAM-1 expression on *ex vivo* expanded Vγ9Vδ2+ T-cells, allowing them to exert greater cytotoxic effects against K562 via PVR and Nectin-2.

## Discussion

γδ T-cells are promising candidates for allogeneic cell therapies due to their anti-tumor activity and ability to function across MHC barriers without causing GvHD (40). While γδ T-cells, expanded *in vivo* or *in vitro* prior to adoptive transfer, have demonstrated anti-tumor activity in preclinical and early phase clinical trials, their efficacy against hematologic malignancies has thus far been modest (12). In this article, we demonstrate that a single session of exercise activates the catecholamine, β2-AR signaling pathway, leading to mobilization of γδ T-cells with enhanced anti-tumor activity into the bloodstream (Supplementary Fig. S3). We further found that γδ T-cells manufactured using exercise or ISO-mobilized cells displayed a cytotoxic phenotype and transcriptomic profile associated with augmented anti-tumor activity *in vitro* and *in vivo*. Inhibiting β2-AR but not β1-AR signaling abrogated the mobilization and *ex vivo* expansion of γδ T-cells with exercise. We also found that an upregulation of the NK-cell cytotoxicity trigger molecule DNAM-1 on γδ T-cells expanded after exercise and β2-AR agonist infusion, and its ability to ligate with the PVR and Nectin-2 expressed by leukemic targets, were directly involved in the anti-tumor response. These findings provide a mechanistic link between exercise, β2-AR activation, and the manufacture of superior γδ T-cell products for adoptive cell therapy against hematologic malignancies (Supplementary Fig. S3).

Exercise has long been known to preferentially mobilize cytotoxic lymphocytes (e.g., NK-cells, CD8^+^ T-cells, γδ T-cells) to the peripheral blood compartment which promptly exit the bloodstream upon cessation of exercise to survey peripheral tissues (37, 41). Recent work has shown that these mobilized cells subsequently infiltrate tumors and are essential in constraining tumor growth in several murine cancer models (42). We recently introduced the innovative concept that lymphocytes mobilized by exercise enhance the graft-versus-leukemia (GvL) effects of donor lymphocyte infusions (35). In addition, an exercise session can improve the *ex vivo* production and effectiveness of virus-specific T-cells, γδ T-cells, and tumor antigen-specific T-cells (43–45). Previously, we reported that exercise augmented the *ex vivo* expansion of γδ T-cells and increased their ability to kill leukemia, lymphoma, and multiple myeloma target cells *in vitro* (10). While an upregulation of the activating receptor NKG2D was found to play a mechanistic role in the increased cytotoxic effects against U266, blocking NKG2D had no affect against K562 (10). Here we found that the activating receptors NKG2D, DNAM-1, TRAIL, CD16, CD56, and CD161, as well as the chemokine receptors CXCR3 and CCR5, were elevated among γδ T-cells expanded after exercise. Furthermore, exercise-expanded γδ T-cells had a lowered expression of the inhibitory receptor NKG2A and a greater proportion of cells with an NKG2D+/NKG2A− phenotype, which is highly predictive of cytotoxic potential (46). Indeed, NKG2A has been touted as a potential candidate for immune checkpoint inhibition (46, 47). The upregulation of DNAM-1 on γδ T-cells expanded after exercise was found to play a mechanistic role in the increased killing of K562 target cells. We found that blocking known DNAM-1 ligands, PVR and Nectin-2, on leukemic targets also abrogated the enhanced effects of exercise on γδ T-cell cytotoxicity. We also observed enhanced cytotoxic activity of exercise-expanded γδ T-cells against ZOL sensitized K562, U266 and Daudi cells. Moreover, our RNA-seq studies revealed an enrichment of gene sets in exercise-expanded γδ T-cells associated with cytokine stimulus and secretion, intracellular and hormone-mediated signaling pathways, antigen presentation and processing, T-cell activation and NK cell–mediated cytotoxicity, among others. It was not surprising, therefore, to find that γδ T-cells expanded after exercise were more capable of restricting K562 leukemic growth in NSG-IL15 mice, particularly when mice received a single dose of ZOL. The enrichment of genes annotated to GO terms such as “cellular response to extracellular stimulus,” may explain why exercise-expanded γδ T-cells were highly responsive to ZOL stimulation both *in vitro* and *in vivo*. Collectively, these findings indicate that exercise-mobilized γδ T-cells develop different phenotypic and transcriptomic characteristics after *ex vivo* expansion, increasing their responsiveness to exogenous antigens and making them more potent killers of multiple hematologic tumors.

The mobilization of γδ T-cells and other effector lymphocytes to blood is largely driven by catecholamines acting on the β2-AR, which is a G protein–coupled receptor that is highly expressed in circulating γδ T-cells (6, 8, 10). At the transcriptional level, we found an enrichment of gene sets involved in G protein–coupled receptor and TOR signaling within exercise-mobilized γδ T-cells, and, within γδ T-cells expanded after exercise, an upregulation of genes involved in cAMP signaling—the major downstream messenger molecule following β2-AR activation. This indicates that exercise-mobilized γδ T-cells are highly sensitive to catecholamines and β2-AR signaling both before and after *ex vivo* expansion. Our interpretations of the single-cell and bulk RNA-seq data were bolstered by our randomized placebo-controlled exercise trial and synthetic β-AR agonist infusion experiments in humans, which clearly demonstrated involvement of the catecholamine- β2-AR signaling axis in the mobilization and expansion of γδ T-cells. First, we showed that a nonselective β1+β2-AR antagonist (nadolol), but not a selective β1-AR antagonist (bisoprolol), administered prior to exercise blocked γδ T-cell mobilization to blood and the subsequent enhanced *ex vivo* expansion. Second, when we infused “resting” participants with ISO—a synthetic β1+β2-AR agonist with approximately 4-fold preference for the β2-AR—for 20 minutes, we were able to expand γδ T-cells from the ISO-mobilized cells that displayed increases in potency and phenotypic and transcriptomic alterations akin to exercise (48). It is noteworthy that the γδ T-cells expanded after ISO infusion controlled leukemic growth in the NSG-IL15 mice receiving ZOL more consistently than those expanded after exercise, possibly due to ISO being infused at a precise dose and rate compared with the inherent variability in endogenous catecholamine responses to fixed intensity acute exercise.

There is increasing evidence that adrenergic signaling is playing a pivotal role in the anti-tumor immune effects provided by exercise. Intermittent “spikes” in catecholamines and other acute exercise factors, such as the muscle-derived cytokines IL6 and IL15, can recruit effector lymphocytes to the blood and facilitate their trafficking to tumors leading to enhanced tumor infiltration and suppression of tumor growth over time (49). In a murine model of melanoma, voluntary wheel running was shown to increase NK-cell tumor infiltration in a manner that was dependent on IL6 and inhibited with nonselective beta blockade (propranolol; ref. 42). More recently, exercise was found to mobilize and accumulate tumor infiltration of IL15-responsive CD8^+^ T-cells in a murine model of pancreatic ductal adenocarcinoma, an effect that was also blocked with propranolol (50). A limitation of these studies is that only a nonselective β1+β2-AR antagonist (propranolol) was used, whereas we have excluded involvement of β1-AR signaling in the mobilization and redistribution of γδ T-cells with exercise by assessing the effects of both selective and nonselective β-AR antagonists. We have also shown previously that virus-specific T-cells, which can exert cytotoxic functions against viral infected tumors (e.g., Epstein-Barr Virus EBV+ lymphoma), are also preferentially redeployed with exercise via the catecholamine- β2-AR signaling axis (44). Collectively, these findings have identified the β2-AR as a therapeutic target that can potentially be exploited to improve anti-tumor immunity, and to mobilize more effective lymphocytes to blood where they can be readily accessed for the *ex vivo* manufacture of more potent cell products for cancer therapy.

In future studies, it will be important to determine whether expanding γδ T-cells with IL2 and IL15 can further augment potency, as it was recently shown that ZOL with IL2 and IL15 enhanced the cytotoxic function of the final γδ T-cell product compared with IL2 alone (51). Moreover, it remains to be seen how repeated γδ T-cell transfers perform in leukemia-bearing mice and to test their potency against a range of hematologic malignancies *in vivo*, both with and without combination therapeutics (e.g., immune checkpoint inhibitors, mAbs) or genetic manipulation (e.g., addition of a chimeric antigen receptor) designed to increase anti-tumor immunity. A minor limitation is our study participants were young (22–41 years), physically active (5–7 PAR score), and aerobically fit (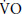
_2max_: 44.9 ± 8.7 mL/kg/minute). It was shown recently that physical fitness is a major determinant of γδ T-cell responsiveness, with successful *ex vivo* γδ T-cell expansions occurring in 100% of physically active donors (physically active >4 days/week) compared with just 25% of sedentary donors (52). It is possible therefore that the augmenting effects of acute exercise on γδ T-cell expansion and potency will be more prominent in sedentary individuals, but this remains to be investigated.

We conclude that exercise-induced β2-AR activation preferentially mobilizes γδ T-cells with an effector phenotype and transcriptomic profile to the peripheral circulation. This allows for the collection and generation of a more potent *ex vivo* expanded γδ T-cell product that is highly effective in killing a broad range of hematologic tumor cells *in vitro and* exert better control of K562 leukemia growth *in vivo*. These findings have significant translational potential for using exercise as a nontoxic and economical therapeutic strategy—or in repurposing existing drugs that target β2-AR signaling—to augment the manufacture of therapeutic cell products for the treatment of refractory and relapsed hematologic malignancies.

## Supplementary Material

Supplementary Figure S1 Supplemental Figure S1: The percentage or MFI of activating, inhibitory, and chemokine receptors among Vd2+ and Vd1+ T-cells before and during acute exercise

Supplementary Figure S2 Supplemental Figure S2: The specific lysis of K562 cells by expanded Vγ9Vδ2+ T-cells with respective blocking of TCR-γδ or CMA.

Supplementary Figure S3 Supplemental Figure S3: Schematic of the overall study design and conclusions.
