# Supplementary material for: Exercise-induced β2-adrenergic Receptor Activation Enhances the Antileukemic Activity of Expanded γδ T-Cells via DNAM-1 Upregulation and PVR/Nectin-2 Recognition
Source: Cancer Res Commun. 2024 May 13;4(5):1253–67. doi: 10.1158/2767-9764.CRC-23-0570 (PMC11090081; doi:10.1158/2767-9764.CRC-23-0570)
Supplement: Supplementary Figure S1 — Supplemental Figure S1: The percentage or MFI of activating, inhibitory, and chemokine receptors among Vd2+ and Vd1+ T-cells before and during acute exercise [file crc-23-0570-s01.pdf]

Supplemental Figure S1

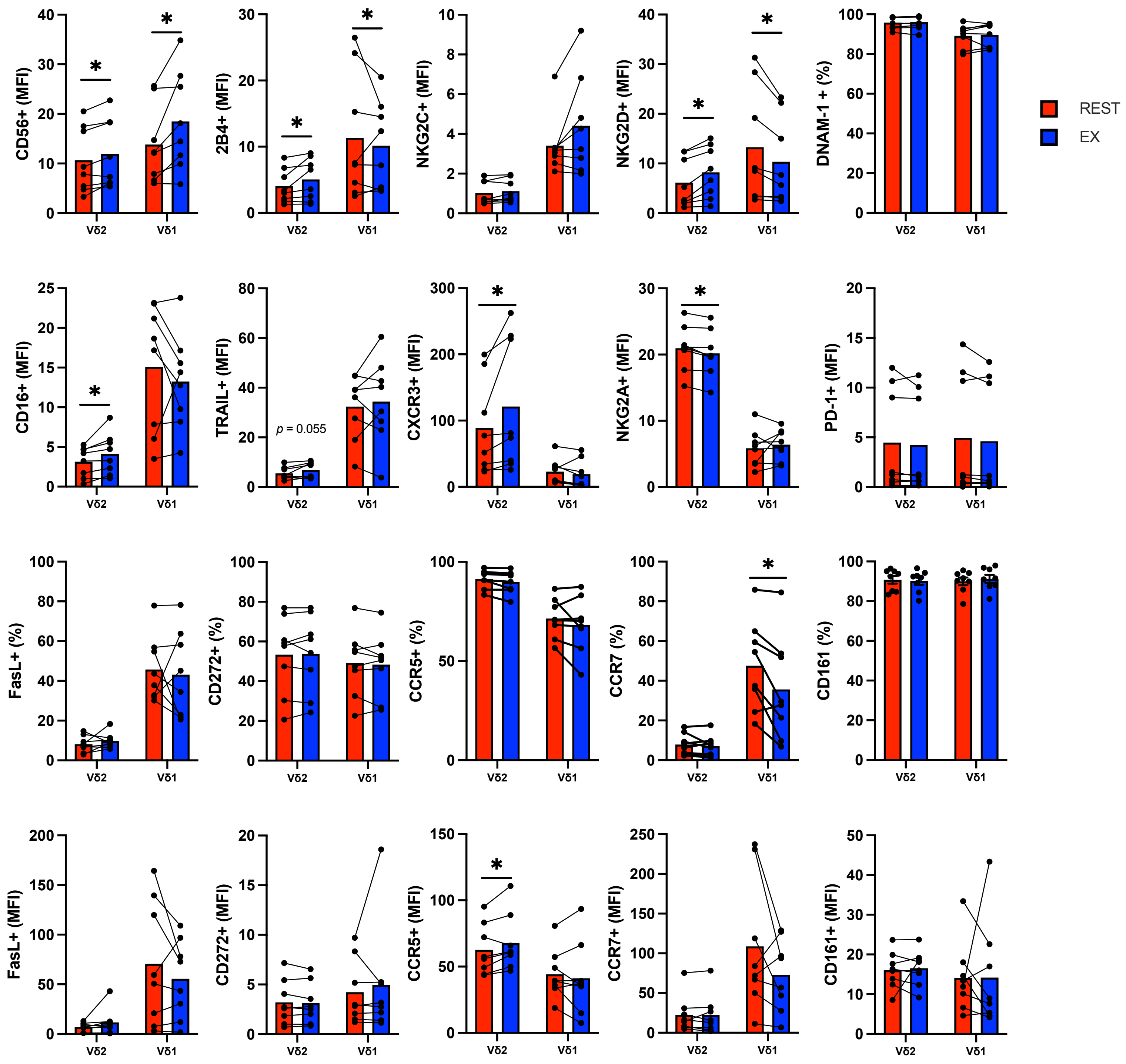

Supplemental Figure S1: The percentage or MFI of activating, inhibitory, and chemokine receptors among Vδ2+ and Vδ1+ T-cells before (REST) and during (EX) acute exercise. (*n*=8). Data are represented as mean and significance is indicated by * ($p < 0.05$); Students two-tailed paired t-test.
