# Supplementary material for: Exercise-induced β2-adrenergic Receptor Activation Enhances the Antileukemic Activity of Expanded γδ T-Cells via DNAM-1 Upregulation and PVR/Nectin-2 Recognition
Source: Cancer Res Commun. 2024 May 13;4(5):1253–67. doi: 10.1158/2767-9764.CRC-23-0570 (PMC11090081; doi:10.1158/2767-9764.CRC-23-0570)
Supplement: Supplementary Figure S2 — Supplemental Figure S2: The specific lysis of K562 cells by expanded Vγ9Vδ2+ T-cells with respective blocking of TCR-γδ or CMA. [file crc-23-0570-s02.pdf]

Supplemental Figure S2

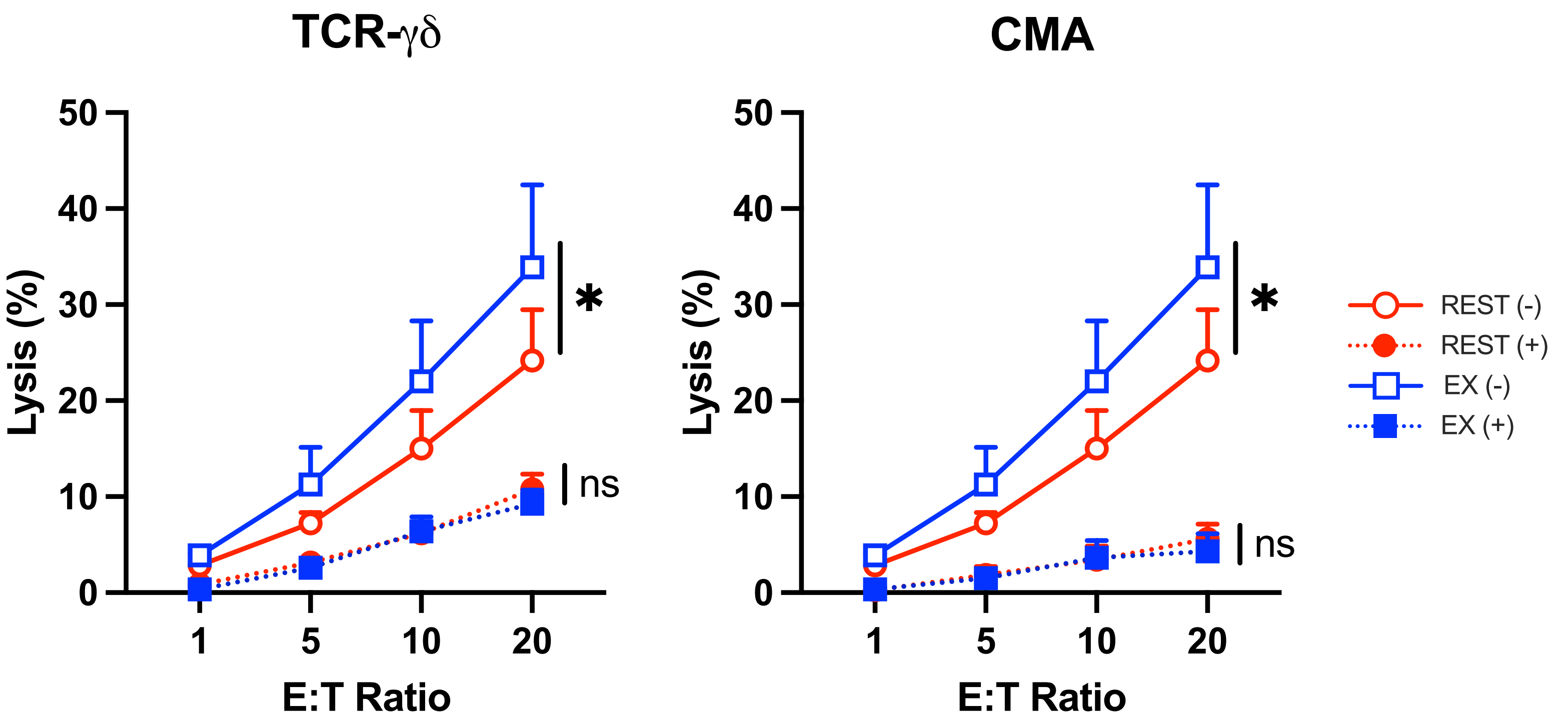

Supplemental Figure S2: The specific lysis of K562 cells by expanded Vγ9Vδ2+ T-cells under two conditions: unstained control (-) and respective blocking antibody or inhibitor (+), TCR-γδ or CMA (*n*=3). Significant differences between the between the unstained (-) and blocking conditions (+) were indicated by * and #, respectively. Data are represented as mean ± SEM; *$p < 0.05$, by repeated measures two-way ANOVA with Bonferroni post hoc test.
