# Supplementary material for: Exercise-induced β2-adrenergic Receptor Activation Enhances the Antileukemic Activity of Expanded γδ T-Cells via DNAM-1 Upregulation and PVR/Nectin-2 Recognition
Source: Cancer Res Commun. 2024 May 13;4(5):1253–67. doi: 10.1158/2767-9764.CRC-23-0570 (PMC11090081; doi:10.1158/2767-9764.CRC-23-0570)
Supplement: Supplementary Figure S3 — Supplemental Figure S3: Schematic of the overall study design and conclusions. [file crc-23-0570-s03.pdf]

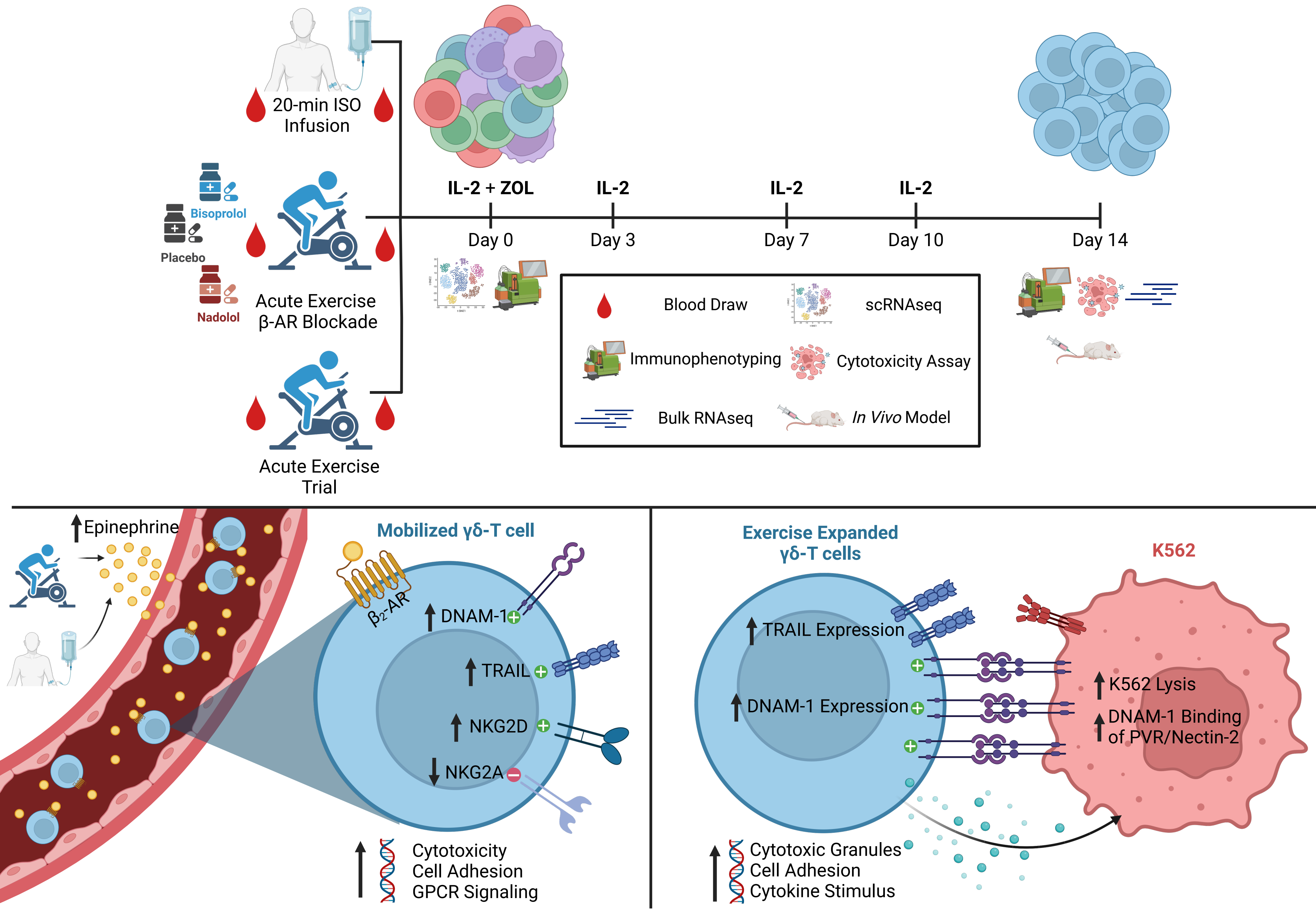
20-min ISO Infusion
Bisoprolol
Placebo
Nadolol
Acute Exercise β-AR Blockade
Acute Exercise Trial
IL-2 + ZOL
IL-2
IL-2
IL-2
Day 0
Day 3
Day 7
Day 10
Day 14
Blood Draw
scRNAseq
Immunophenotyping
Cytotoxicity Assay
Bulk RNAseq
In Vivo Model
Epinephrine
Mobilized γδ-T cell
β2-AR
DNAM-1
TRAIL
NKG2D
NKG2A
Cytotoxicity
Cell Adhesion
GPCR Signaling
Exercise Expanded γδ-T cells
K562
TRAIL Expression
DNAM-1 Expression
K562 Lysis
DNAM-1 Binding of PVR/Nectin-2
Cytotoxic Granules
Cell Adhesion
Cytokine Stimulus

**Supplemental Figure S3**: Schematic of the overall study design and conclusions. Exercise activates the catecholamine-β2-AR signaling pathway preferentially mobilizing γδ T-cells to the peripheral circulation with an effector phenotype and transcriptomic profile, which allows for the generation of a potent *ex vivo* expanded γδ T-cell product that is highly effective in killing a broad range of hematologic tumor cells in vitro and exert better control of K562 leukemia growth *in vivo*. Importantly, the upregulation of the DNAM-1 on γδ T-cells expanded after exercise and its ability to ligate with the PVR and Nectin-2 expressed by leukemic targets, were directly involved in the anti-tumor response.
